# Escape of a Tænia Solium through the Umbilicus

**Published:** 1845-11

**Authors:** 


*Escape of a Tænia solium through the Umbilicus.* By M. Siebold.—In April, 1841, a man, 22 years of age, was received into the hospital, labouring under scrofulous abscesses in various parts of the body. A little above the umbilicus there existed a considerable deposit of softened scrofulous matter, and a small abscess, which opened immediately over the umbilicus, gave it the appearance of that of a new-born child. One day something was felt moving within this abscess over the umbilicus, and was found to consist of a loop of the intestinal worm, the *Tænia solium.* This loop appeared to be endowed with life, was white, but no trace of chylous or excrementitial matter was observed. On pulling out this loop one extremity was discovered to become more slender, as if near the head, and on pulling at this end the entire head of the worm was drawn out. The tail portion was afterwards extracted with ease by means of gentle traction. The worm was several yards long, and when put into lukewarm water, moved about and appeared quite healthy. No gas or feculent matter escaped from the aperture through which it escaped, nothing, in fact, which could make it be supposed that the animal had come through an intestinal perforation. No bad symptoms followed this escape of the tænia. The probe could not detect any communication with the intestine, on account of the acute pain which such exploration caused. The patient died about a year afterwards, but leave to open the body could not be procured.—*Ibid, from Arch. Gen. de Med.*

